# Editorial: Exercise-induced neuroplasticity in neurodegeneration diseases, volume II

**DOI:** 10.3389/fnins.2025.1746951

**Published:** 2025-12-04

**Authors:** Laikang Yu, Lingxiao He, Yih-Kuen Jan

**Affiliations:** 1 Beijing Key Laboratory of Sports Performance and Skill Assessment, Beijing Sport University, Beijing, China; 2 Department of Sports Performance, Beijing Sport University, Beijing, China; 3 School of Public Health, Xiamen University, Xiamen, China; 4 Department of Health and Kinesiology, University of Illinois Urbana-Champaign, Urbana, IL, United States

**Keywords:** exercise, physical activity, neuroplasticity, cognitive function, neurodegeneration diseases

## Introduction

Neurodegenerative diseases, including Alzheimer's disease (AD), Parkinson's disease (PD), and related disorders, represent an escalating global health challenge. As life expectancy increases, the burden of chronic neurological conditions continues to rise (Imam et al., 2025). Despite extensive research efforts, the development of effective pharmacological disease-modifying therapies remains limited. As a result, growing attention has been directed toward non-pharmacological strategies aimed at preventing, delaying, or mitigating neurodegeneration. Among such strategies, exercise has emerged as one of the most promising interventions due to its capacity to modulate neuroplasticity—the brain's intrinsic ability to reorganize and adapt through changes in structure, function, and connectivity (Erickson et al., 2011; Northey et al., 2018).

Exercise-induced neuroplasticity encompasses a broad spectrum of adaptive mechanisms, including synaptic remodeling, angiogenesis, mitochondrial biogenesis, and enhanced neurogenesis within brain regions critical for cognition and motor control (Liang et al., 2021; Lu et al., 2023; Zhang et al., 2024). These neurobiological adaptations contribute to improved neural efficiency, resilience, and repair, counteracting pathological processes such as oxidative stress, neuroinflammation, and protein aggregation that characterize neurodegenerative disorders (Li et al., 2024; Lu et al., 2023; Sun et al., 2022). Recent evidence suggests that the beneficial effects of exercise extend beyond cellular and molecular levels to encompass network-level plasticity, facilitating improved communication among distributed brain regions (Dimitriadis et al., 2024; Rosso et al., 2025; Yu et al., 2024; Zhang et al., 2022). Such findings position exercise not only as a therapeutic tool for symptom management but also as a potential modifier of disease progression.

Building on the success of the first volume of this Research Topic, the second volume continues to expand our understanding of the mechanisms and practical applications of exercise in the context of neurodegenerative diseases.

## Summary of selected articles from this Research Topic

Thirteen manuscripts were received for this Frontiers Research Topic. After rigorous review, 4 articles were finally accepted for publication. The contributing 32 authors were from 5 countries, including China, Qatar, Türkiye, Tunisia, and Romania. This Research Topic received more than 27,000 views and downloads as of November 2025. The key contents and findings of each paper are as follows:

Shao et al. conducted a comprehensive meta-analysis evaluating the impact of Tai Chi on cognitive performance in individuals with mild cognitive impairment (MCI). Synthesizing evidence from nine randomized controlled trials (RCTs), the authors reported significant improvements in global cognitive function and memory following Tai Chi interventions. Notably, the magnitude of cognitive enhancement varied by Tai Chi style, with 24-form and 10-form routines demonstrating distinct advantages across cognitive domains. These findings support Tai Chi as a promising, low-cost, and culturally adaptable mind-body intervention for mitigating cognitive decline and promoting neuroplasticity in older adults with MCI.

Wan et al. presented an insightful opinion article proposing an innovative diagnostic framework for MCI that integrates motion performance assessment with electroencephalography (EEG). They argued that, while gait and balance analyses provided valuable behavioral indicators of early cognitive decline, combining these measures with EEG-derived neural features substantially enhanced diagnostic precision. This multimodal approach captures both motor and neural signatures of cognitive dysfunction, offering a more holistic understanding of MCI pathophysiology. The authors further highlighted the promise of dual-task paradigms and machine learning techniques in refining this strategy, paving the way for more sensitive, non-invasive, and personalized tools for early MCI detection.

Ishaq et al. conducted a comprehensive systematic review examining how different exercise regimens influence molecular mechanisms within the nigrostriatum in animal models of Parkinson's disease (PD). Their analysis indicated that exercise mitigates neuroinflammation and neuronal apoptosis by suppressing α-synuclein accumulation and inflammatory signaling cascades, while simultaneously enhancing neurotrophic support through increased brain-derived neurotrophic factor (BDNF) and glial cell line-derived neurotrophic factor (GDNF) expression. Additionally, exercise promoted dopaminergic signaling and synaptic integrity, collectively contributing to neuroprotection and improved motor function. These findings underscored the multifaceted molecular benefits of exercise and implied its therapeutic potential in delaying PD progression.

Ben Ezzdine et al. provided an extensive review clarifying how diverse physical activity modalities foster neuroplasticity and cognitive resilience in individuals with neurodegenerative disorders. Their synthesis demonstrated that aerobic, resistance, mind-body, and dual-task exercises engage distinct yet complementary biological pathways, including enhanced neurotrophic signaling, reduced neuroinflammation and oxidative stress, and strengthened synaptic connectivity. These interventions yielded measurable improvements in memory, executive function, and emotional regulation. The authors also emphasized emerging artificial intelligence (AI)-driven, personalized exercise strategies as a promising frontier for optimizing cognitive rehabilitation and tailoring interventions to individual neurological profiles.

Collectively, the studies included in this second volume of Exercise-Induced Neuroplasticity in Neurodegeneration Diseases reinforce the transformative potential of exercise as both a preventive and therapeutic strategy for neurodegenerative diseases. The diverse approaches—ranging from traditional mind-body practices such as Tai Chi, to mechanistic animal studies on PD, to innovative diagnostic frameworks integrating EEG and motion analysis—highlight the multidimensional effects of exercise on the brain. These contributions converge on a central theme: exercise modulates neuroplasticity through intertwined molecular, structural, and functional pathways, ultimately enhancing cognitive and motor resilience in the context of neurodegeneration.

Notably, the increasing focus on individualized and technology-driven interventions represents a promising advance toward precision neurorehabilitation. The integration of AI, multimodal neuroimaging, and physiological monitoring enables tailored exercise prescriptions based on individual neurobiological profiles, thereby optimizing outcomes across different stages of disease. Moreover, the exploration of culturally adaptable and accessible exercise modalities, such as Tai Chi, addresses the global imperative for equitable and sustainable approaches to brain health promotion.

As the field advances, future research should prioritize longitudinal, mechanistic, and translational studies to bridge preclinical findings with clinical applications. Expanding interdisciplinary collaborations among neuroscientists, clinicians, and exercise physiologists will be critical to fully leverage the neuroprotective potential of exercise. Ultimately, this Research Topic underscores that exercise is not merely an adjunct intervention but a central driver of neuroplastic adaptation—enhancing synaptic connectivity, supporting neural network integrity, and promoting functional resilience. Through these mechanisms, exercise contributes to preserving brain function, slowing neurodegenerative processes, and fostering healthy cognitive aging.
